# Comparison of the therapeutic effects of 15 mg and 30 mg initial daily prednisolone doses in patients with subacute thyroiditis: a multicenter, randomized, open-label, parallel-controlled trial

**DOI:** 10.1080/07853890.2023.2288941

**Published:** 2023-12-04

**Authors:** Jingjing Zeng, Aihua Jia, Juan Zhang, Bin Gao, Jing Xu, Ying Xing, Xiaorui Jing, Yang Jiao, Jie Wang, Wenlei Xu, Ling Gao, Lei Shang, Shaoyong Xu

**Affiliations:** a Department of Endocrinology, Xiangyang Central Hospital, Affiliated Hospital of Hubei University of Arts and Science, Xiangyang, Hubei, China; b Center for Clinical Evidence-Based and Translational Medicine, Xiangyang Central Hospital, Affiliated Hospital of Hubei University of Arts and Science, Xiangyang, China; c Department of Endocrinology, No.1 Hospital of Yulin, Yulin, China; d Department of Endocrinology, 3201 Hospital of Xi’an Jiao tong University Health Science Center, Hanzhong, Shaanxi, China; e Department of Endocrinology, Tangdu Hospital, Air Force Medical University, Shaanxi, China; f Department of Endocrinology, The Second Affiliated Hospital of Xi’an Jiaotong University, Xi’an, China; g Department of Endocrinology, Daxing Hospital, Xi’an, China; h Department of Health Statistics, Shaanxi Key Laboratory of Free Radical Biology and Medicine and the Ministry of Education Key Lab of Hazard Assessment and Control in Special Operational Environment, School of Public Health, Air Force Medical University, Xi’an, Shaanxi, China

**Keywords:** Initial dose, prednisolone, subacute thyroiditis, randomized controlled trial

## Abstract

**Introduction:**

Current guidelines recommendations for the initial dose of prednisolone (PSL) in the treatment of subacute thyroiditis (SAT) are based on low-quality studies. We designed a randomized controlled trial (RCT) to compare the efficacy and safety of using a low initial dose of PSL with a standard initial dose of PSL in SAT patients.

**Patients and methods:**

This open-label RCT was conducted at five hospitals in China from June 2019 to January 2022. SAT patients with moderate-to-severe pain or a poor response to non-steroidal anti-inflammatory drugs (NSAIDs) were randomly assigned in a 1:1 ratio to the experimental and control groups. The initial dose of PSL was 15 mg/d in the experimental group and 30 mg/d in the control group. The primary outcome was the total duration of PSL treatment, with non-inferiority prespecified with a margin of 7 days. Clinical trial registration number: ChiCTR1900023884.

**Results:**

The full analysis set included 60 patients (30 in each group). The mean duration of PSL treatment in the experimental and control group was 34.62 ± 14.12 and 41.18 ± 16.89 days, respectively, meeting the non-inferiority criterion (p_non-inferiority_ = 0.0006). The total dose of PSL used in the experimental group was lower than in the control groups (330 vs 595 mg, *p* < 0.0001). There were no differences in the mean time to pain relief and complete resolution, the occurrence of recurrence, hypothyroidism, or adverse events between the groups.

**Conclusions:**

The initial dose of 15 mg/d of PSL was not inferior to the dose of 30 mg/d in terms of efficacy and showed a similar safety profile. A low initial dose of PSL could be recommended for Chinese adult SAT patients who have a suboptimal response using NSAIDs or experience moderate-to-severe pain.

**KEY MESSAGES**
1. Low initial dose (15 mg/d) of prednisolone was non-inferior to the standard initial dose of prednisolone (30 mg/d) in treatment duration, time to pain relief, or the prevalence of hypothyroidism, recurrence, and adverse reactions in the treatment of subacute thyroiditis.
2. Patients with subacute thyroiditis administered a low initial dose of prednisolone had a lower total dose of prednisolone compared to those receiving the standard dose of prednisolone.

## Introduction

Subacute thyroiditis (SAT), also known as granulomatous thyroiditis or de Quervain thyroiditis, is an inflammatory disease of the thyroid gland and the most common cause of pain in the thyroid gland [1,2]. The main clinical symptom of SAT is neck pain, which can radiate to the area behind the ears, jaw, occipital region, and throat [3]. SAT is a self-limiting disease. The primary goal of initial treatment is to use non-steroidal anti-inflammatory drugs (NSAIDs) or glucocorticoids to relieve pain symptoms, followed by a reduction in the risk of SAT recurrence and permanent hypothyroidism.

The American Thyroid Association (ATA) and Chinese Society of Endocrinology (CSE) recommend the use of glucocorticoids for rapid symptom relief when NSAIDs treatment is ineffective (absence of symptom remission after 2–3 days of treatment) or when the patients initially present with moderate-to-severe pain (based on the physician decision to relieve the pain immediately) [4–6]. The alternative to prednisolone (PSL) in terms of glucocorticoids such as prednisone which is preferred in many hospitals. The ATA recommends an initial dose of 40 mg/d of PSL, which should be gradually tapered off and discontinued completely within six weeks following symptom relief [4]. The CSE guidelines recommend an initial PSL dose of 20–40 mg/d [5]. Even though these are strong recommendations, they are based on low-quality evidence [3,7–11]. There is a lack of high-quality studies on the optimal initial dose of PSL for SAT treatment.

There are various short-term and long-term side effects of glucocorticoids on multiple organs, such as hyperglycemia, Cushing’s syndrome, obesity, arrhythmia, gastrointestinal bleeding or ulcers, osteoporosis, cataracts, and sepsis [12–15]. Additionally, these side effects are dependent on the initial dose and cumulative dose of the glucocorticoid administered [16]. Hence, seeking a low initial dose and shortening the regimen duration while ensuring an optimal therapeutic effect is important. Three studies have explored the feasibility of a strategy using a low initial dose of PSL [17–19]. For example, Kubota S et al. [17] conducted a pioneering single-arm study, which showed that a treatment strategy with a low initial dose of PSL (15 mg/d) and a reduction of 5 mg every 2 weeks was safe and effective in a Japanese population [17]. Unfortunately, these studies were retrospective or observational, and well-designed randomized controlled trials (RCT) to determine a low initial dose of PSL have not been conducted.

Therefore, the aim of this study was to compare the efficacy and safety of using a low initial dose of PSL (15 mg/d) with a standard initial dose of PSL (30 mg/d) in SAT patients who were unresponsive to NSAIDs or had moderate-to-severe pain.

## Materials and methods

### Study design

An investigator-initiated, multicenter, randomized, open, parallel-controlled trial was conducted from June 2019 to January 2022 at five hospitals in China: (1) Xiangyang Central Hospital, Affiliated Hospital of Hubei University of Arts and Science (Xiangyang, Hubei); (2) 3201 Hospital of Xi’an Jiao Tong University Health Science Center (Xi’an, Shaanxi); (3) Number 1 Hospital of Yulin (Yulin, Shaanxi); (4) Tangdu Hospital, Air Force Medical University (Xi’an, Shaanxi); (5) Xi’an Daxing Hospital (Xi’an, Shaanxi). The study is reported according to CONSORT guidelines in Appendix A (Supplementary Material).

The study protocol was approved (XYSZXYY-LLDD-PJ-2019-032) by the Ethics Committee of Xiangyang Central Hospital and was aligned with the Declaration of Helsinki 1964 and its later amendments. All participants provided written informed consent. The protocol has been registered (ChiCTR1900023884) in the clinical registry of China and has been published in advance [20].

### Participants

SAT patients aged 18–70 years with moderate-to-severe pain (visual analog scale (VAS) score up to 7/10) or a poor response to oral NSAIDs (no pain relief within 3 days of treatment) met the inclusion criteria. The diagnostic criteria for SAT and the exclusion criteria are provided in Appendix B and Appendix C, respectively (Supplementary Material).

Eligible individuals were recruited consecutively. During the screening process, demographic characteristics, physical examination, medical history, and medication history were recorded in face-to-face interviews. Furthermore, samples of blood and urine were collected for laboratory tests. VAS and a five-point Likert scale were used to measure pain in the thyroid gland. Radioisotope scanning, radioactive iodine uptake tests, and fine-needle aspiration cytology were used to assist in SAT diagnosis.

Patients who met the inclusion criteria were randomly assigned to the experimental group or the control group within 2 days after the screening process. Body weight and laboratory data at the screening stage were taken to be baseline values. Vital signs, thyroid-pain score, and physical examination of the thyroid gland were measured again as baseline values at the time of randomization. The details of the trial procedures are described in Appendix D (Supplementary Material).

### Randomization and masking

The eligible individuals were randomly assigned in a 1:1 ratio. Block randomization was employed to stratify participants according to the research center. Using the ‘PROC PLAN’ procedure statement in SAS 9.4, given the number of seeds and setting a block length to 4, a random coding table of 92 individuals was generated. All randomization groups were numbered, subdivided, and retained by a third party (Lei Shang) who was not involved in data collection. The generation of random codes was undertaken by an external programmer not involved in the RCT. When eligible individuals from one center had been enrolled, the researchers communicated with the third party to inquire about the numbers and the corresponding treatment plans. This RCT was an open-label study. We needed to adjust the medication based on the treatment response of patients during the RCT. Therefore, researchers and patients could not be blinded to the study protocol: only the laboratory staff and data analysts were blinded to the study protocol.

### Interventions

The eligible patients were randomly assigned to either the experimental group, receiving a low initial dose, or the control group, receiving a standard initial dose of PSL. Patients in both groups were treated with PSL (p.o.) for several weeks to months. The discontinuation of PSL is gradually implemented based on clinical symptoms, laboratory test results, and principles of dose reduction. Patients were assessed once or twice daily (usually in a hospital) during the first week after randomization and followed up every 2–4 weeks for up to 6 months.

In the experimental group, patients initially received a dose of 15 mg/d of PSL for 14 days. Subsequently, the dose was gradually reduced by 5 mg every 7–14 days or longer, which was based on previous literature [17], the expertise of senior clinicians, and our preliminary study conducted on a small sample size. In the control group, the initial dose of PSL was 30 mg/d, and the reduction process began 3–7 days after complete pain resolution (self-rated pain score = 0, absence of tenderness in the thyroid gland). The dose was decreased by 5–10 mg every 5–7 days, following guideline recommendations [5]. Considering that most patients would receive complete pain resolution within 5–10 days, patients received 30 mg/d of PSL in the control group for approximately 5–14 days. However, patients in the experimental group received a fixed initial dose of 15 mg/d of PSL for 14 days, irrespective of the time taken for complete pain resolution. Dosage adjustments in both groups were made based on the subject’s response to treatment, adhering to the aforementioned principles. Furthermore, as a non-inferiority study, if there is no recurrence, the treatment period for both groups would be approximately 4 weeks.

Researchers were cautious about prescribing other medications needed for the safety and health of patients. Combined treatments and other treatment measures are detailed in Appendix E (Supplementary Material). Termination of treatment with PSL was considered in cases where subjects showed a poor response to PSL (Appendix F, Supplementary Material).

### Outcomes

The primary outcome measure was the total duration of PSL treatment, including the duration of continued treatment after dose reduction or withdrawal of PSL. Secondary outcome measures were the time to pain relief (defined as a reduction in pain severity >50%), complete resolution time of pain, the total dose of PSL, relapse, hypothyroidism, and changes in laboratory parameters such as thyroid function. ‘Relapse’ was defined as the reappearance of pain symptoms after their disappearance, including relapse during a reduction in the PSL dose or after withdrawal of PSL. ‘Hypothyroidism’ was defined as a thyroid-stimulating hormone (TSH) level beyond the upper limit of normal upon treatment cessation or during follow-up. Levothyroxine was given if TSH >10 mU/L. Serious adverse events are defined in the Appendix G (Supplementary Material).

### Statistical analyses

The sample size was calculated based on the total duration of PSL treatment. Alpha (one-sided) = 0.05 with a non-inferiority margin of 7 days was employed assuming that the treatment duration in the experimental group was identical to that in the control group, with a standard deviation (SD) of 12 days [17,19]. We calculated that having 38 patients in each group could achieve a statistical power of 80%. The sample size was determined to be 90 assuming 20% of patients would be lost to follow-up. In July 2020, we revised the protocol to add an interim analysis when the sample size reached 2/3, which was approved by the Ethics Committee of Xiangyang Central Hospital (XD-2020-013). An interim analysis comprising 60 participants who completed the trial by January 2022 was conducted. The ‘true’ difference in treatment duration between the two groups was −6.56 days (SD was 14.12 and 16.89 days, respectively). A sample size of 60 could achieve a statistical power >95% upon data analyses. Due to the non-inferiority results shown in the interim analysis, we decided to terminate the trial early, which was approved by the Ethics Committee of Xiangyang Central Hospital (JT-2022-001) and the data monitoring committee.

For the efficacy measures, including both primary and secondary outcomes, analyses were undertaken on the full analysis set. In the case of missing indicators of outcomes, multiple imputation methods were used to fill in the missing values [21]. The results of the per-protocol analysis were reported as a supplement. The safety outcomes were evaluated based on safety-set analysis.

For continuous variables, mean and SD were used for data with a normal distribution, while median and interquartile range were used for variables with a non-normal distribution (P25, P75). Student’s *t*-test and Wilcoxon rank-sum test were employed to compare differences between groups. For categorical variables, percentages were used, and between-group comparisons were performed using the chi-square test or Fisher’s exact test.

The principal endpoint was assessed by estimating the between-group difference in treatment duration using Student’s *t*-test and checking it against the pre-defined non-inferiority margin of 7 days. In the sensitivity analysis for the primary outcome measures, covariance analysis was used to control for the influence of the study centers. For secondary outcome measures, the generalized linear model was utilized to control for the study centers. For comparison of the changes in blood parameters before and after treatment, a generalized linear model was used to correct the baseline values. Statistical analyses were conducted using SAS 9.4 software (SAS Institute, Cary, NC, USA). *p* < 0.05 was considered statistically significant, and the α level for the first interim analysis was set at 0.005 considering the inflation of Type I errors caused by multiple analyses.

## Results

### Participants and characteristics at baseline

**Figure 1.**
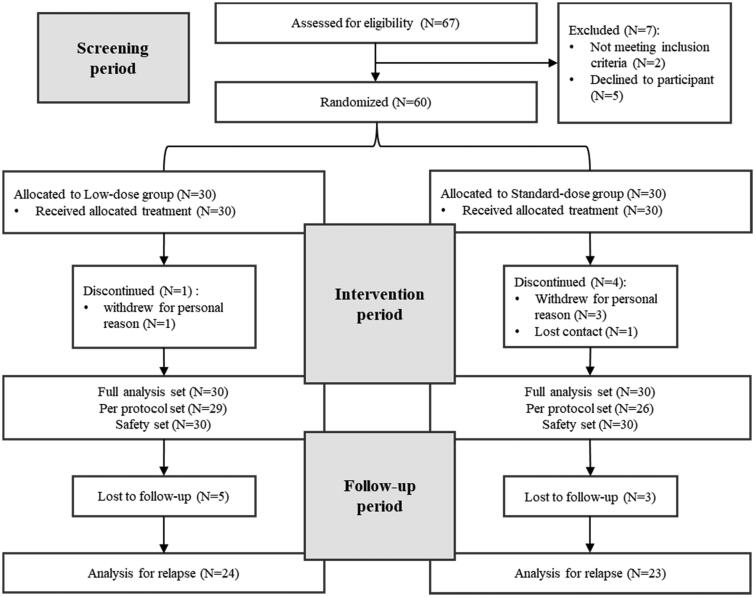
Flowchart of the randomized controlled trial.

A total of 67 SAT patients underwent eligibility screening. Seven patients failed the screening, and 60 patients were randomized into two groups with 30 patients in each group. During the treatment, 1 participant in the experimental group withdrew from the study due to personal reasons while 3 participants in the control group withdrew from the study due to personal reasons, and 1 participant was lost to follow-up. Furthermore, there were 5 patients in the experimental group and 3 patients in the control group who were lost to follow-up after discontinuation (Figure 1).

In the experimental group, the majority of patients (more than 80%) reduced dose in the following sequence: 15 mg/d–10 mg/d–5 mg/d–withdrawal, while the minority of patients reduced dose from 15 mg/d to 5 mg/d directly or from 5 mg/d to 2.5 mg/d and then withdrawal. In the control group, half of the patients received an initial dose of 30 mg/d followed by a reduction of 5 mg each time. The other half received an initial dose of 30 mg/d and then reduced to 20 mg/d directly, followed by a reduction of 5 mg or 10 mg each time. The actual reduction process of PSL for patients with and without recurrence for both groups was described in Appendix H (supplementary material).

The mean age of patients was 49.84 ± 11.89 years, and 33.33% of them were male. The median self-reported pain score (VAS) was 7. There were no significant differences between the experimental group and control group for demographic characteristics, pretreatment with NSAIDs, pain in the thyroid gland, vital signs, or laboratory parameters at baseline (Tables 1 and 2). The initial ‘dose/body weight’ ranged between 0.21–0.35 mg/kg and 0.36–0.64 mg/kg respectively in the experimental group and the control group. More than 85% of patients in the experimental group had a dose/body weight range of 0.21–0.30 mg/kg, while in the control group, over 85% of patients had a range of 0.41–0.60 mg/kg.

**Table 1.** Baseline characteristics of the study participants.

	Total	Low-dose group (*N* = 30)	Standard-dose group (*N* = 30)	*p*-value
Age (year), mean (SD)	49.84 ± 11.89	49.32 ± 12.57	50.37 ± 11.33	0.735
Gender (male), *n* (%)	20 (33.33%)	9 (30.00)	11 (36.67)	0.525
BMI (kg/m^2^)	22.89 ± 2.62	22.28 ± 2.24	23.50 ± 2.86	0.080
Education, *n* (%)				0.772
High school or below	47 (79.66)	25 (83.33)	22 (75.86)	
Junior college	5 (8.47)	2 (6.67)	3 (10.34)	
College or above	7 (11.86)	3 (10.00)	4 (13.79)	
Smoking, *n* (%)	5 (8.20)	2 (6.67)	3 (10.00)	0.671
Alcohol drinking, *n* (%)	10 (16.67)	5 (16.67)	5 (16.67)	>0.999
Pretreatment with NSAIDs, *n* (%)	15 (25.00)	9 (30.00)	6 (20.00)	0.371
Time of pain before treatment (days)	27 (13, 34)	29 (12, 45)	23 (14, 31)	0.181
Respiratory tract infection, *n* (%)	32 (53.33)	14 (46.67)	18 (60.00)	0.246
VAS, median (P_25_, P_75_)	7 (6, 8)	7 (6, 8)	7 (4, 8)	0.740
Likert, median (P2_5_, P_75_)	3 (3, 4)	3 (3, 4)	3 (2, 4)	0.685
Hypermetabolism, *n* (%)	52 (86.67)	27 (90.00)	25 (83.33)	0.732
Fever, *n* (%)	44 (75.86)	22 (73.33)	22 (78.57)	0.503
Heart rate, mean (SD)	92.86 ± 12.11	90.77 ± 12.68	95.26 ± 11.18	0.161
Respiratory rate, median (P_25_, P_75_)	20 (19, 20)	20 (19, 20)	20 (19, 20)	0.961
Systolic pressure, median (P_25_, P_75_)	120 (111, 130)	126 (111, 135)	117.0 (111.0, 125.5)	0.092
Diastolic pressure, mean (SD)	76.78 ± 10.62	76.90 ± 10.39	76.64 ± 11.07	0.926
Goiter, *n* (%)	48 (80.00)	28 (93.33)	20 (66.67)	0.052
Thyroid nodule, *n* (%)	20 (33.33)	9 (30.00)	11 (36.67)	0.584
Thyroid tenderness, *n* (%)	58 (98.31)	30 (100.00)	28 (96.55)	0.492

Values were presented as mean ± standard deviation for normally distributed variables, median (interquartile range) for non-normal data, or number (percentage) for categorical variable.

SD: standard deviation; NSAIDs: non-steroidal anti-inflammatory drugs; VAS: visual analogue scale.

**Table 2.** Biochemical parameters of participants at baseline.

	Total	Low-dose group(*N* = 30)	Standard-dose group (*N* = 30)	*p*-value
TT3 (nmol/L)	3.17 (2.37, 4.33)	3.13 (2.34, 4.31)	3.26 (2.78, 4.38)	0.436
TT4 (nmol/L)	207.17 ± 69.99	198.71 ± 75.14	215.96 ± 64.49	0.375
TSH (uIU/mL)	0.02 (0.01, 0.05)	0.02 (0.01, 0.03)	0.01 (0.01, 0.16)	0.956
FT3 (pmol/L)	9.27 (6.46, 13.10)	9.06 (6.71, 10.86)	10.07 (6.18, 14.30)	0.494
FT4 (pmol/L)	34.97 (26.16, 50.73)	32.20 (26.16, 46.70)	35.55 (27.09, 50.83)	0.622
TPOAB (IU/mL)	14.60 (6.20, 21.09)	14.76 (4.58, 18.27)	13.77 (8.97, 27.37)	0.804
TRAB (IU/mL)	1.03 (0.43, 13.15)	0.89 (0.38, 9.67)	1.33 (0.67, 13.28)	0.323
CRP (mg/L)	54.42 ± 36.58	49.30 ± 39.45	58.97 ± 33.94	0.351
ESR (mm/h)	67.53 ± 25.12	65.60 ± 27.96	69.84 ± 21.58	0.538
HB (g/L)	120 (112, 128)	121 (112, 127)	118 (113, 128)	0.755
RBC (10^12^/L)	4.15 (3.90, 4.43)	4.14 (3.90, 4.37)	4.17 (3.88, 4.55)	0.687
WBC (10^9^/L)	7.61 (5.55, 8.70)	7.06 (5.31, 8.40)	8.06 (6.89, 9.64)	0.052
PLT (10^9^/L)	300 (253, 361)	290 (253, 321)	306 (253, 384)	0.117
AST (U/L)	17.0 (14.0, 24.0)	16.10 (12.50, 25.25)	18 (15, 22)	0.277
ALT (U/L)	19.0 (13.0, 34.0)	17.5 (11.5, 38.2)	19.3 (14.0, 31.0)	0.866
ALP (U/L)	96.5 (81.0, 114.0)	85.5 (64, 125)	97.5 (85.0, 114.0)	0.409
TB (μmol/L)	10.6 (8.4, 13.7)	10.85 (8.70, 14.55)	10.2 (7.2, 13.1)	0.454
SCr (μmol/L)	47.3 (41.3, 57)	48.3 (40.7, 59.0)	46.40 (41.55, 56.45)	0.887
BUN (mmol/L)	4.20 (3.30, 5.00)	4.20 (3.60, 5.50)	4.17 (3.10, 4.94)	0.199
FBG (mmol/L)	5.28 ± 0.85	5.46 ± 0.89	5.12 ± 0.80	0.164

Values were presented as mean ± standard deviation for normally distributed variables, median (interquartile range) for non-normal data. TT3: total triiodothyronine; TT4: total thyroxine; TSH: thyroid stimulating hormone; FT3: free triiodothyronine; FT4: free thyroxine; TPOAb: anti-thyroid peroxidase; TRAb: thyrotrophin receptor antibody; CRP: C-reactive protein; ESR: erythrocyte sedimentation rate; HB: hemoglobin; RBC: red blood cell count; WBC: white blood cell count; PLT: platelet count; AST: aminotransferase; ALT: alanine transaminase; ALP: alkaline phosphatase; TB: total bilirubin; SCr: serum creatinine; BUN: blood urea nitrogen; FBG: fasting blood-glucose.

### Primary outcomes

**Figure 2.**
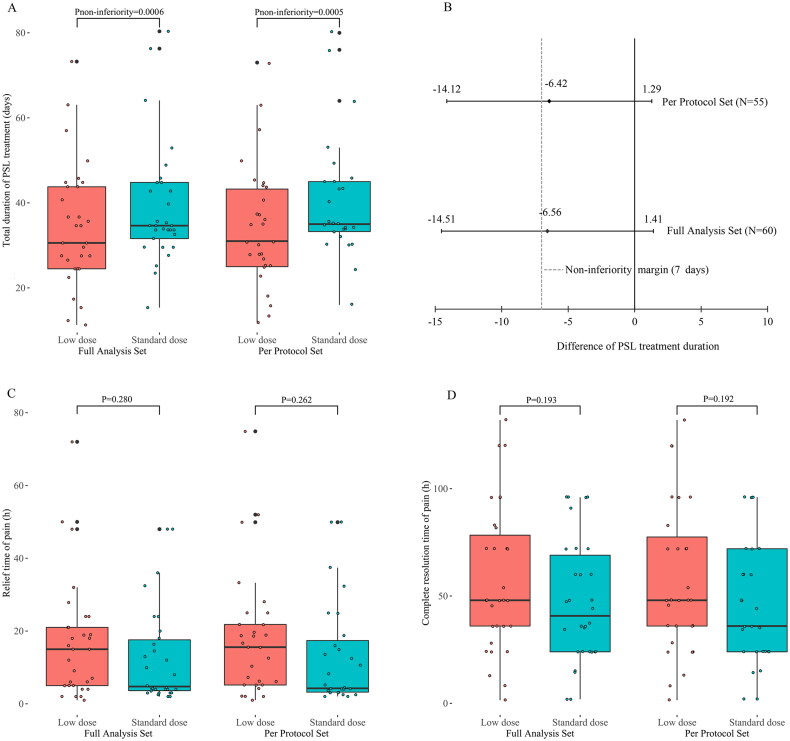
Comparison of the total duration of prednisolone treatment, relief time of pain, and complete resolution time of pain between the Low dose group and the Standard dose group. (A) the total duration of prednisolone treatment (days) for patients with subacute thyroiditis. (B) Differences between groups (Low-dose group – Standard-dose group) and non-inferiority testing for the total duration of prednisolone treatment. (C) the relief time of thyroid pain (h) for patients with subacute thyroiditis. (D) the complete resolution time of thyroid pain (h) for patients with subacute thyroiditis. PSL: prednisolone.

The mean duration of PSL treatment was 34.62 ± 14.12 days in the experimental group and 41.18 ± 16.89 days in the control group, resulting in a difference of −6.56 ± 15.55 days between the two groups. The non-inferiority test showed that the duration of PSL treatment was non-inferior in the experimental group compared with that in the control group (with a noninferiority margin of 7 days, p_non-inferiority_ = 0.0006) (Figure 2). Analysis of covariance adjusted for the study centers showed no significant difference in treatment duration between the groups, with a between-group difference of −5.09 days (*p* = 0.200).

The sensitivity analysis showed that the difference in the duration of PSL treatment between the two groups was −6.42 ± 14.35 (34.20 ± 14.17 *vs*. 40.62 ± 14.55) days, and the p-value for the non-inferiority test was 0.0005 in the per-protocol set, consistent with the full analysis set. Covariance analysis in the per-protocol set showed no significant change after adjustment for the study centers, with a between-group difference of −4.77 days (*p* = 0.202). There was no difference in the duration of PSL treatment between patients with or without NSAIDs pretreatment (*p* = 0.188).

### Secondary outcomes

The full analysis set revealed no significant differences in the mean time required for pain relief and complete resolution between the experimental group and control group (16.80 ± 15.91 *vs*. 12.68 ± 13.35 h, *p* = 0.280; 57.68 ± 33.55 *vs*. 47.14 ± 28.69 h, *p* = 0.193, respectively). The PSL dose did not follow a normal distribution. The median dose of PSL was 330 mg in the experimental group and 595 mg in the control group. The Wilcoxon rank-sum test showed that patients in the experimental group received a lower PSL dose compared to the control group (*p* < 0.0001) (Supplementary Figure 1). The sensitivity analysis results showed that the results in the per-protocol set were consistent with the full analysis set (Figure 2). There were no significant differences in pain relief time, complete resolution time, or total dose of PSL between participants with or without pretreatment with NSAIDs (*p* = 0.667, 0.107, and 0.898, respectively).

The prevalence of recurrence was 16.67% in the experimental group and 13.04% in the control group, and the difference between the two groups was not significant (*p* = 0.727). There were no significant differences between the recurrence group and no recurrence group for demographic characteristics, and clinical features at baseline (Table 3).

**Table 3.** Baseline characteristics of the participants with or without recurrence.

	Recurrence(*N* = 7)	No recurrence (*N* = 40)	*p*-value
Age (year), mean (SD)	50.57 ± 10.64	50.47 ± 11.83	0.984
Gender (male), *n* (%)	1 (14.29)	16 (40.00)	0.396
BMI (kg/m^2^)	23.06 ± 2.12	22.64 ± 2.57	0.722
Education, *n* (%)			0.597
High school or below	5 (83.33)	32 (82.05)	
Junior college	0 (0.00)	3 (7.69)	
College or above	1 (16.67)	4 (10.26)	
Smoking, *n* (%)	0 (0.00)	3 (7.50)	>0.999
Alcohol drinking, *n* (%)	1 (14.29)	7 (17.50)	0.912
Pretreatment with NSAIDs, *n* (%)	1 (14.29)	9 (22.50)	0.520
Time of pain before treatment (days)	18 (14, 26)	28 (13, 34)	0.750
Respiratory tract infection, *n* (%)	2 (28.57)	21 (52.50)	0.416
VAS, median (P_25_, P_75_)	7 (5, 7)	8 (7, 8)	0.137
Likert, median (P2_5_, P_75_)	3 (2, 4)	3 (3, 4)	0.492
Hypermetabolism, *n* (%)	7 (100.00)	34 (85.00)	0.571
Fever, *n* (%)	5 (71.43)	29 (74.36)	>0.999
Heart rate, mean (SD)	98.50 ± 9.03	91.92 ± 12.64	0.229
Respiratory rate, median (P_25_, P_75_)	20 (20, 21)	20 (19, 20)	0.181
Systolic pressure, median (P_25_, P_75_)	124 (111, 134)	121 (117, 130)	0.947
Diastolic pressure, mean (SD)	80.00 ± 10.74	77.67 ± 11.40	0.626
Goiter, *n* (%)	4 (57.14)	33 (82.50)	0.155
Thyroid nodule, *n* (%)	1 (14.29)	12 (30.77)	0.654
Thyroid tenderness, *n* (%)	7 (100.00)	39 (97.50)	>0.999

Values were presented as mean ± standard deviation for normally distributed variables, median (interquartile range) for non-normal data, or number (percentage) for categorical variable.

SD: standard deviation; VAS: visual analogue scale.

**Figure 3.**
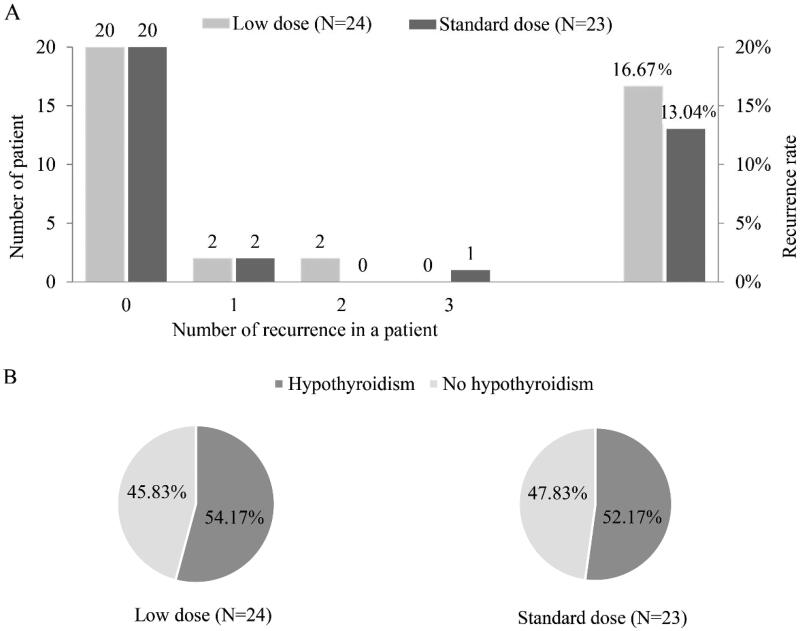
Thyroid pain recurrence and hypothyroidism for subacute thyroiditis patients with prednisolone treatment in the low-dose group and the standard-dose group. (A) Recurrence time and rate. (B) Incidence of hypothyroidism.

There was no significant difference in the prevalence of hypothyroidism (54.17% vs 52.17%, *p* = 0.891) (Figure 3). The difference in changes in biochemical parameters before and after treatment of PSL between groups was not significant (Table 4).

**Table 4.** Changes of biochemical parameters before and after treatment of prednisolone ^a^.

	Low-dose group	Standard-dose group	*p*-value	Adjusted *p*-value^b^
TT3 (nmol/l)	−1.47 (–2.76, −0.70)	−2.58 (–3.23, −1.17)	0.264	0.108
TT4 (nmol/L)	−121.86 ± 106.10	−142.86 ± 80.76	0.607	0.490
TSH (uIU/mL)	2.86 (0.17, 14.18)	5.03 (3.12, 9.16)	0.642	0.574
FT3 (pmol/L)	−5.38 (–8.87, −2.80)	−4.74 (–8.43, −2.65)	0.678	0.251
FT4 (pmol/L)	−21.90 (–45.75, −15.60)	−26.25 (–40.39, −17.86)	0.757	0.720
CRP (mg/L)	−32.42 (–58.10, −26.69)	−60.94 (–83.80, −30.04)	0.412	0.271
ESR (mm/h)	−59.48 ± 21.11	−60.22 ± 20.70	0.912	0.492
HB (g/L)	11.00 (5.00, 18.00)	17.0 (5.0, 25.0)	0.175	0.134
RBC (10^12^/L)	0.42 ± 0.36	0.53 ± 0.35	0.358	0.222
WBC (10^9^/L)	−0.08 ± 2.28	−1.47 ± 2.54	0.090	0.756
PLT (10^9^/L)	−71.29 ± 50.82	−81.75 ± 57.13	0.563	0.365
AST (U/L)	5.85 (–1.20, 11.00)	1.0 (–3.0, 4.0)	0.102	0.983
ALT (U/L)	1.5 (–10.0, 11.0)	−1.0 (–6.0, 4.0)	0.338	0.644
ALP (U/L)	−17.36 ± 25.16	−17.40 ± 31.60	0.998	0.764
TB (μmol/L)	−0.02 ± 6.06	−0.03 ± 5.05	0.998	0.640
SCr (μmol/L)	8.97 ± 6.55	5.20 ± 8.42	0.202	0.174
BUN (mmol/L)	−0.06 ± 0.97	0.54 ± 1.18	0.164	0.277
FBG (mmol/L)	−0.20 (–0.63, 0.33)	−0.21 (–0.68, 0.47)	0.903	0.370

^a^ Biochemical parameters were not filled in.

^b^ *p*-value derived from the analysis based on generalized linear model adjusting for baseline value.

Values were presented as mean ± standard deviation for normally distributed variables or median (interquartile range) for non-normal data.

TT3: total triiodothyronine; TT4: total thyroxine; TSH: thyroid stimulating hormone; FT3: free triiodothyronine; FT4: free thyroxine; CRP: C-reactive protein; ESR: erythrocyte sedimentation rate; HB: hemoglobin; RBC: red blood cell count; WBC: white blood cell count; PLT: platelet count; AST: aminotransferase; ALT: alanine transaminase; ALP: alkaline phosphatase; TB: total bilirubin; SCr: serum creatinine; BUN: blood urea nitrogen; FBG: fasting blood-glucose.

### Safety outcomes

No serious adverse reactions were reported. Only 13.33% of patients experienced mild adverse reactions, such as palpitation, skin symptoms, gastrointestinal symptoms, facial edema, and so on. There was no significant difference in adverse reactions between the two groups (13.33% *vs*. 13.33%) (Table 5).

**Table 5.** Adverse reaction.

	Low-dose (*N* = 30)	Standard-dose (*N* = 30)	*p*
Total adverse reaction	4 (13.33)	4 (13.33)	>0.999
Palpitation and syncope	0	1	
Limb edema	1	0	
Facial edema	0	1	
Insomnia	0	1	
Hoarseness and pruritus	0	1	
Weight gain	1	0	
Stomach pain	1	0	
Hyperglycemia	1	0	

## Discussion

This is the first multicenter RCT to evaluate the efficacy and safety of using a low initial dose of PSL for the treatment of moderate-to-severe SAT. The results demonstrate that the low initial dose (15 mg/d) of PSL was non-inferior to the standard initial dose of PSL (30 mg/d) in treatment duration, time to pain relief, or the prevalence of hypothyroidism, recurrence, and adverse reactions. Patients administered a low initial dose of PSL had a lower total dose of PSL compared to those receiving the standard dose of PSL.

We have validated the conclusions of retrospective cohort studies which suggested that a low initial dose (15 mg/d) of PSL may achieve better outcomes compared to a high initial dose [18]. The mean treatment duration was 34.62 and 41.18 days in the experimental group and control group, respectively, with 90% of patients completing treatment within 8 weeks, which is similar to those of other studies. For example, treatment durations with an initial PSL dose of 30–40 mg/d has been reported to be 34–52.6 days [3,7,8]. A single-arm study from Japan including 219 patients showed a median treatment duration of 42 days with an initial PSL dose of 15 mg/d, and over 80% of patients received treatment for ≤8 weeks [17]. Our study is also important in terms of pain relief and complete pain resolution as most previous studies have been retrospective and lacked data on these parameters [17–19]. We showed that approximately 90% of patients achieved symptom remission within 24 h, and over 80% of patients experienced complete pain resolution within 72 h, with no differences between the two groups. Hence, PSL can relieve pain symptoms effectively even if the initial dose is 15 mg/d [19].

In SAT patients, transient hypothyroidism may occur during the disease course, with 5%–26% of patients experiencing permanent hypothyroidism [3,22,23]. We found that 39.19% of patients had a TSH level >10 mU/L and were treated with drugs. Benbassat et al. reported that 60% of SAT patients had hypothyroidism, with 39% of SAT patients having a TSH level >10 mU/L [24]. However, the prevalence of hypothyroidism has also been reported inconsistently across studies. Kubota et al. showed a hypothyroidism prevalence in 34.54% of patients administered an initial PSL dose of 15 mg/d [17]. Li et al. showed the prevalence of transient hypothyroidism was 4.8% after an initial PSL dose of 20 mg/d [25]. The discrepancy between these results and our data may be due to (i) our patients having more severe symptoms; (ii) other studies potentially using NSAIDs in combination; (iii) differences in study designs. Additionally, we revealed no significant difference in the prevalence of recurrence of pain symptoms between the two groups (16.67% and 13.04%), which was lower than that reported in studies using a standard initial dose of PSL (20%–35%) [9,11,26]. Another retrospective study showed a prevalence of recurrence of 15% in SAT patients treated with PSL, with the initial dose of PSL not influencing the prevalence of recurrence of SAT [27]. In this study, considering the possibility of insufficient anti-inflammatory effects, the initial dose in the low-dose group was maintained for a sufficient duration to reduce the risk of recurrence. Furthermore, as seen in the actual reduction process, the low initial dosage group had a longer duration in the 5 mg/d phase before drug withdrawal.

Our study has four main limitations. Firstly, approximately 20% of patients dropped out during the treatment period or were lost to follow-up. However, most of the dropouts were due to personal reasons rather than poor treatment outcomes or adverse reactions. Multiple imputation method was utilized to fill in the missing values, and the per-protocol set was used to validate the stability of our results. Secondly, patients were followed up for only 6 months after withdrawal of PSL, which limited our ability to determine whether the prevalence of hypothyroidism was transient or permanent. However, we found that the proportion of patients with short-term hypothyroidism and a TSH level >10 mU/L was similar between the two groups. Hence, the initial dose of PSL may only have a slight effect on the risk of permanent hypothyroidism. Thirdly, no serious adverse reactions were reported during the treatment and follow-up period. We found that 13.33% of patients had mild adverse reactions, which is consistent with the findings of Li et al.’s study [25]. Although there was no significant difference in the prevalence of adverse reactions between the two groups, the short duration of follow-up prevented us from determining the long-term effect of different initial doses of PSL on adverse effects. The experimental group had a lower initial dose and lower total dose of PSL, suggesting a potential association with fewer long-term adverse effects from a theoretical perspective [16]. Finally, since the majority of SAT patients were followed up by telephone and biochemical and clinical parameters were not measured during treatment, we did not show the time response of these parameters to the gradual reduction of prednisolone dose.

## Conclusions

A low initial dose of PSL (15 mg/d) was not inferior to the standard dose of PSL (30 mg/d) in terms of efficacy and showed a similar safety profile. The use of a low initial dose of PSL could be recommended for Chinese adult SAT patients with suboptimal response to NSAIDs or moderate-to-severe pain. Further studies with longer follow-up periods in multiple countries are needed to validate our findings.

## Supplementary Material

Supplemental Material

## Data Availability

The data that support the findings of this study are available from the corresponding author, Shaoyong Xu, upon reasonable request.
