# Supplementary material for: Comparison of the therapeutic effects of 15 mg and 30 mg initial daily prednisolone doses in patients with subacute thyroiditis: a multicenter, randomized, open-label, parallel-controlled trial
Source: Ann Med. 2023 Dec 4;55(2):2288941. doi: 10.1080/07853890.2023.2288941 (PMC10836262; doi:10.1080/07853890.2023.2288941)
Supplement: Supplemental Material [file IANN_A_2288941_SM9415.zip › Supplementary Appendix.docx]

**Appendix A** CONSORT 2010 checklist of information to include when reporting a randomised trial

| Section/Topic | Item No | Checklist item | Reported on page No |
| --- | --- | --- | --- |
| Title and abstract | | | |
|  | 1a | Identification as a randomised trial in the title | 1 |
|  | 1b | Structured summary of trial design, methods, results, and conclusions (for specific guidance see CONSORT for abstracts) | 3 |
| Introduction | | | |
| Background and objectives | 2a | Scientific background and explanation of rationale | 4 |
|  | 2b | Specific objectives or hypotheses | 4 |
| Methods | | | |
| Trial design | 3a | Description of trial design (such as parallel, factorial) including allocation ratio | 5 |
|  | 3b | Important changes to methods after trial commencement (such as eligibility criteria), with reasons | 7 |
| Participants | 4a | Eligibility criteria for participants | 5 |
|  | 4b | Settings and locations where the data were collected | 5 |
| Interventions | 5 | The interventions for each group with sufficient details to allow replication, including how and when they were actually administered | 6-7 |
| Outcomes | 6a | Completely defined pre-specified primary and secondary outcome measures, including how and when they were assessed | 7 |
|  | 6b | Any changes to trial outcomes after the trial commenced, with reasons | - |
| Sample size | 7a | How sample size was determined | 7 |
|  | 7b | When applicable, explanation of any interim analyses and stopping guidelines | 7 |
| Randomisation: |  |  |  |
| Sequence generation | 8a | Method used to generate the random allocation sequence | 6 |
|  | 8b | Type of randomisation; details of any restriction (such as blocking and block size) | 6 |
| Allocation concealment mechanism | 9 | Mechanism used to implement the random allocation sequence (such as sequentially numbered containers), describing any steps taken to conceal the sequence until interventions were assigned | 6 |
| Implementation | 10 | Who generated the random allocation sequence, who enrolled participants, and who assigned participants to interventions | 6 |
| Blinding | 11a | If done, who was blinded after assignment to interventions (for example, participants, care providers, those assessing outcomes) and how | 6 |
|  | 11b | If relevant, description of the similarity of interventions | - |
| Statistical methods | 12a | Statistical methods used to compare groups for primary and secondary outcomes | 7-8 |
|  | 12b | Methods for additional analyses, such as subgroup analyses and adjusted analyses | 8 |
| Results | | | |
| Participant flow (a diagram is strongly recommended) | 13a | For each group, the numbers of participants who were randomly assigned, received intended treatment, and were analysed for the primary outcome | 7-8 |
|  | 13b | For each group, losses and exclusions after randomisation, together with reasons | Figure1 |
| Recruitment | 14a | Dates defining the periods of recruitment and follow-up | 8 |
|  | 14b | Why the trial ended or was stopped | 7 |
| Baseline data | 15 | A table showing baseline demographic and clinical characteristics for each group | Table1 |
| Numbers analysed | 16 | For each group, number of participants (denominator) included in each analysis and whether the analysis was by original assigned groups | 8 |
| Outcomes and estimation | 17a | For each primary and secondary outcome, results for each group, and the estimated effect size and its precision (such as 95% confidence interval) | 9-10 |
|  | 17b | For binary outcomes, presentation of both absolute and relative effect sizes is recommended | 9-10 |
| Ancillary analyses | 18 | Results of any other analyses performed, including subgroup analyses and adjusted analyses, distinguishing pre-specified from exploratory | 9-10 |
| Harms | 19 | All important harms or unintended effects in each group (for specific guidance see CONSORT for harms) | - |
| Discussion | | | |
| Limitations | 20 | Trial limitations, addressing sources of potential bias, imprecision, and, if relevant, multiplicity of analyses | 11-12 |
| Generalisability | 21 | Generalisability (external validity, applicability) of the trial findings | 12 |
| Interpretation | 22 | Interpretation consistent with results, balancing benefits and harms, and considering other relevant evidence | 10-12 |
| Other information | | |  |
| Registration | 23 | Registration number and name of trial registry | 5 |
| Protocol | 24 | Where the full trial protocol can be accessed, if available | 5 |
| Funding | 25 | Sources of funding and other support (such as supply of drugs), role of funders | 3 |

**Appendix B** The diagnostic criteria of subacute thyroiditis

1. Acute onset, with most upper respiratory infections occurring within 1-2 weeks prior to onset.

2. The thyroid gland is painful to palpation and may have radiating pain in the ear and jaw angle. The thyroid gland is often enlarged, diffusely or asymmetrically with mild/moderate enlargement, with or without nodules, and has a hard texture.

3. It may be accompanied by fever (mostly low or moderate), chills, chills, fatigue, loss of appetite, palpitations, and hand tremors.

4. Thyroid function is hyperactive.

5. Decreased 24h iodine uptake rate of the thyroid gland or thyroid nuclide test suggesting "non-visible or vaguely visible thyroid gland".

6. Significantly elevated erythrocyte sedimentation rate (ESR) or C-reactive protein (CRP).

7. Ultrasound of the thyroid gland indicates a lamellar hypoechoic shadow at the site of the lesion.

8. Negative or very low levels of thyrotropin receptor antibody (TRAb) and thyroid peroxidase antibody (TPO-Ab). Thyroglobulin (Tg) levels are significantly elevated, consistent with the degree of thyroid destruction.

9. Normal or high white blood cell count.

10. A fine needle aspiration cytology (FNAC) of the thyroid gland reveals multinucleated giant cells.

11. Other thyroid disorders can be excluded.

Of these, 4-5 must be present to suggest bidirectional segregation in combination with other suggestive signs to confirm SAT. If 5 are absent, 10 must be present. FNAC is not a routine test for SAT, but FNAC can be used to diagnose SAT directly.

**Appendix C** The exclusion criteria of the study

Subjects should not enter the study if any of the following exclusion criteria are fulfilled:

1. women who are pregnant, intending to become pregnant during the study period, currently lactating females, or women of child-bearing potential not using highly effective, medical approved birth control methods.

2. Suspicion or diagnosis of: a. Acute suppurative thyroiditis; b. Graves’ disease; c. Hashimoto thyroiditis; d. Thyroid carcinoma.

3. Glucocorticoids allergy or intolerance (e.g. systemic allergic reaction, induced asthma, bleeding, ulcer and perforation of stomach or intestine).

4. Previous treatment with PSLs in the last 6 months prior to screening.

5. Adrenocortical hyperfunction, uncontrolled hypertension, and diabetes.

6. Patients with clinically apparent liver disease characterized by either one of the following:

a. ALT or AST > 3x upper limit of normal confirmed on two consecutive measurements (by local laboratory) within 4 weeks prior to screening period

b. Impaired excretory (e.g. hyperbilirubinemia) and/or synthetic function, or other conditions of decompensated liver disease such as coagulopathy, hepatic encephalopathy, hypoalbuminemia, ascites and bleeding from oesophageal varices.

c. Acute viral or active autoimmune, alcoholic, or other types of hepatitis.

7. Patients with moderate /severe renal impairment or end-stage renal disease (estimated Glomerular Filtration Rate ≤ 60 mL/min calculated by using the abbreviated equation developed by the Modification of Diet in Renal Disease study with modification for the Chinese population) at screening or within 4 weeks prior to screening (by local laboratory).

8. Congestive heart failure defined as New York Heart Association class III or IV.

9. Significant cardiovascular history within the past 3 months prior to screening defined as: myocardial infarction, coronary angioplasty or bypass graft(s), valvular disease or repair, unstable angina pectoris, transient ischemic attack, or cerebrovascular accident.

10. History of mental illness.

11. History of corneal ulcer.

12. History of gastrointestinal disease including gastroenterostomy, enterectomy, Roemheld Syndrome, severe hernia, and intestinal obstruction.

13. Postoperative patients with unhealing wound.

14. Diagnosed and/or treated malignancy (except for basal cell skin cancer, in situ carcinoma of the cervix, or in situ prostate cancer) within the past 5 years.

15. History of organ transplant or acquired immunodeficiency syndrome.

16. History of alcohol abuse or illegal drug abuse within the past 12 months.

17. Potentially unreliable patients and those judged by the investigator to be unsuitable for the study.

**Appendix D** The trial procedures

| **Study Period** | **Screening** | **Intervention period** | | | | |
| --- | --- | --- | --- | --- | --- | --- |
| **Visit number** | 1 | 2 | 3* | 4-x | x | x + n |
| **Study interval** | -0-3d | Randomization | 1w | every 2-4 w | PSL discontinuation | every 2-4w |
| **Visit window** |  |  | (±2d) | (±2d) | (±2d) | (±7d) |
| **Telephone visit** |  |  |  | √ |  |  |
| **Screening/Demography/Baseline** |  |  |  |  |  |  |
| Written informed consent | √ |  |  |  |  |  |
| Inclusion/Exclusion criteria | √ | √ |  |  |  |  |
| Demographics | √ |  |  |  |  |  |
| Physical examination, height, and weight | √ |  |  |  |  |  |
| Medical/ Current conditions | √ |  |  |  |  |  |
| History of diabetes & complications | √ |  |  |  |  |  |
| **Intervention** |  |  |  |  |  |  |
| Instruction of drugs |  | √ | √ | √ | √ | √ |
| Thyroid pain (VAS) | √ | √ | √ | √ | √ |  |
| Thyroid tenderness | √ | √ | √ | √ | √ |  |
| Temperature monitor | √ | √ | √ | √ | √ |  |
| CRP and ESR | √ |  | √ |  | √ |  |
| Thyroid function | √ |  |  |  | √ | √ |
| 24h I-131 uptake or thyroid scintigraphy | √ |  |  |  |  |  |
| Hematology panel | √ |  |  |  | √ |  |
| Liver function | √ |  |  |  | √ |  |
| Creatinine, Uric acid | √ |  |  |  | √ |  |
| Glucose | √ |  |  |  | √ |  |
| Adverse events |  | √ | √ | √ | √ | √ |

* The patients will receive face-to-face daily visit within the first week in research centers, and thyroid pain, temperature and adverse events will be assessed in each visit. VAS: visual analog scales; ESR: erythrocyte sedimentation rate; CRP: C-reactive protein.

The subjects were assessed once or twice a day (usually in a hospital) during the first week after randomization, including self-reported pain scores, thyroid tenderness, body temperature, heart rate, and adverse events. The time of significant pain relief (over 50% reduction in pain severity) and the time of complete pain relief were recorded. CRP, ESR and thyroid function were measured in the first week after randomization. Telephone follow-up was carried out at each scheduled drug reduction date to determine the change of the condition and the occurrence of adverse events, and record and guide the patients to use the drug. If there were no relapses, the subjects were given a dose reduction. In case of recurrence, such as aggravation of pain or reappearance of pain that has disappeared, the subject is advised to come to the hospital for detailed evaluation and treatment according to the condition, for example, delay the original dose of PSL for a long time or increase the dose of PSL appropriately (5-10mg is recommended). The lowest maintenance dose (5 mg) of PSL was given for 3-7 days. If the patient has no thyroid pain and the CRP and ESR levels are normal, the patient can stop the drug, otherwise the PSL treatment time will be prolonged. If recurrence occurs after discontinuation of the drug, the treatment shall be carried out according to the above-mentioned principle of treatment of recurrence after reduction of the drug.

**Appendix E** Combined treatments and other treatment measures

During the treatment period, anti-ulcer drugs will be administered to some patients with history of intestinal ulcer or who feel uncomfortable in stomach during treatment, and hepatologist will be consulted for possible antiviral therapy in patients with inactive hepatitis. In the present study, anti-thyroid drugs will have no role and beta-blockers will be used as needed to control thyrotoxic symptoms. Levothyroxine will be employed when patients have a TSH level above 10 mIU/L or symptoms during the hypothyroid stage. In addition, although viral infection is regarded as the possible cause of SAT, anti-viral medications will not be suggested in the study.

**Appendix F** Trial termination

According to the clinical experience and the results of the pre-experiment, the pain of most patients will disappear completely within 5-10 days after receiving PSL treatment, no matter the low initial dose or the standard initial dose. Therefore, if the test group still has pain at two weeks, consider termination, adjust to the standard initial dose for treatment and specify the reason (with the exception of the situation that the pain completely disappears but the symptoms recur subsequently). In addition, no matter in the low initial dose or in the standard initial dose group, if the pain does not relieve significantly three days after PSL is given, it is necessary to re-evaluate the SAT diagnosis, eliminate other causes (such as acute thyroiditis) and give corresponding treatment if confirmed; if other causes are excluded, the study can be considered to be terminated, and the low initial dose can be adjusted to the standard initial dose for treatment and record the reasons in detail, the standard initial dose can be increased to a higher dose (such as 40mg/d) and specify the reason.

**Appendix G** Definition of serious adverse effects

serious adverse event means any adverse event that led to any of the following:

(a) death,

(b) serious deterioration in the health of the subject, that resulted in any of the following:

(i) life-threatening illness or injury,

(ii) permanent impairment of a body structure or a body function,

(iii) hospitalisation or prolongation of patient hospitalisation,

(iv) medical or surgical intervention to prevent life-threatening illness or injury or permanent impairment to a body structure or a body function,

(v) chronic disease,

(c) foetal distress, foetal death or a congenital physical or mental impairment or birth defect.

**Appendix H** The actual reduction process of PSL in experimental group and control group.

In the experimental group, the majority (more than 80%) of patients reduced dose in the order of 15mg/d‒10mg/d‒5 mg/d‒withdrawal, with a small number of patients reduced dose from 15mg/d to 5mg/d directly or from 5mg/d to 2.5mg/d and then withdrawal. In the population who reduced dose normally (without recurrence), the initial dose of 15 mg/d lasted for a mean of 13.23 ± 3.32 days, with a median (inter-quartile range) of 14 (12, 15) days; the dose of 10 mg/d lasted for a mean of 8.65 ± 2.64 days, with a median (inter-quartile range) of 8 (7, 10) days; and the dose of 5 mg/d lasted for a mean of 9.45 ± 5.23 days, with a median (inter-quartile range) of 8 (7, 21) days. 4 patients suffered relapses. One patient occurred during the reduction from 15 mg/d to 10 mg/d. One patient occurred twice during the reduction from 15 mg/d to 10 mg/d and from 10 mg/d to 5 mg/d. One patient relapsed after PSL withdrawal. Remarkably, one patient had a severe recurrence of pain during the second reduction from 15 mg/d to 10 mg/d and was given a standard dose of 30 mg/d and then treated according to the reduction principle of the standard initial dosage group.

In the control group, half of the patients received an initial dose of 30 mg/d followed by a reduction of 5 mg each time. Half received an initial dose of 30 mg/d and then reduced to 20 mg/d directly, followed by a reduction of 5 mg or 10 mg each time. In the population without recurrence, the initial dose of 30 mg/d lasted for a mean of 8.25 ± 2.83 days, with a median (inter-quartile range) of 8 (7, 8) days; the dose of 25 mg/d lasted for a mean of 6.33 ± 2.50 days, with a median (inter-quartile range) of 6 (4, 7) days; the dose of 20 mg/d lasted for a mean of 6.40 ± 2.76 days, with a median (inter-quartile range) of 7 (4, 7) days; the dose of 15 mg/d lasted for a mean of 5.84 ± 1.46 days, with a median (inter-quartile range) of 6 (5, 7) days; the dose of 10 mg/d lasted for a mean of 6.82 ± 2.86 days, with a median (inter-quartile range) of 7 (5, 7) days; the dose of 5 mg/d lasted for a mean of 7.00 ± 3.99 days, with a median (inter-quartile range) of 7 (4, 9) days. 3 patients suffered relapses. One patient occurred during the reduction from 15 mg/d to 10 mg/d. One patient occurred once during the reduction from 15 mg/d to 10 mg/d, and twice after the PSL withdrawal. Another relapse occurred during the initial dose, which was adjusted to 35 mg/d for 4 days and then continued with 30 mg/d, followed by the reduction strategy in the standard dose group.
