# Supplementary material for: Comparison of the therapeutic effects of 15 mg and 30 mg initial daily prednisolone doses in patients with subacute thyroiditis: a multicenter, randomized, open-label, parallel-controlled trial
Source: Ann Med. 2023 Dec 4;55(2):2288941. doi: 10.1080/07853890.2023.2288941 (PMC10836262; doi:10.1080/07853890.2023.2288941)
Supplement: Supplemental Material [file IANN_A_2288941_SM9415.zip › Supplementary Materials.docx]

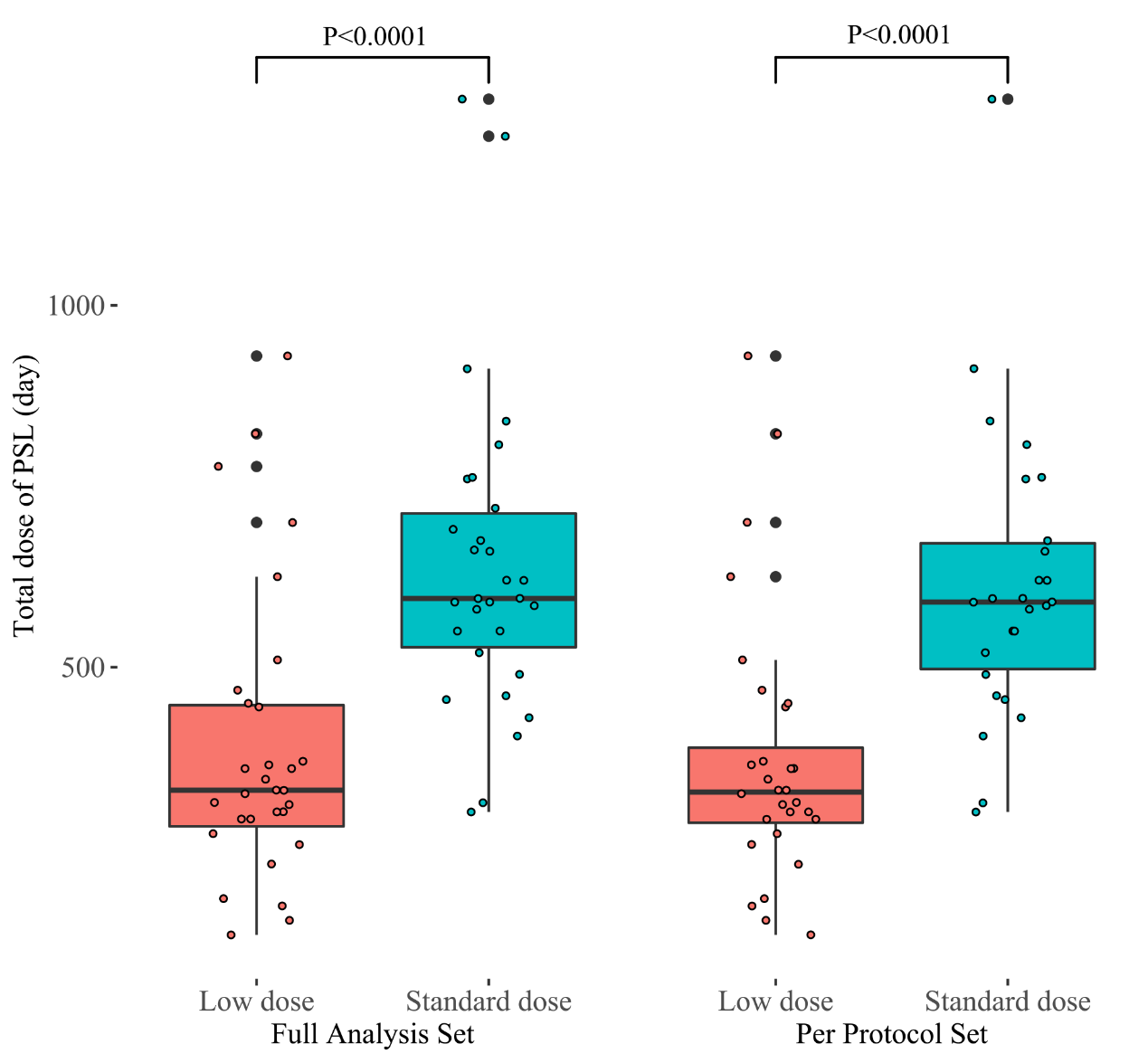


**Supplementary Figure 1** Total dose of prednisolone for subacute thyroiditis patients in the low-dose group and the standard-dose group. PSL: prednisolone.
